# Severe chronic non-bacterial osteomyelitis in combination with total MPO deficiency and responsiveness to TNFα inhibition

**DOI:** 10.3389/fimmu.2023.1233101

**Published:** 2023-10-26

**Authors:** Martina Sundqvist, Karin Christenson, Per Wekell, Halla Björnsdottir, Agnes Dahlstrand Rudin, Felix P. Sanchez Klose, Tilmann Kallinich, Amanda Welin, Lena Björkman, Johan Bylund, Anna Karlsson-Bengtsson, Stefan Berg

**Affiliations:** ^1^ Department of Rheumatology and Inflammation Research, Institute of Medicine, Sahlgrenska Academy University of Gothenburg, Gothenburg, Sweden; ^2^ Department of Oral Microbiology and Immunology, Institute of Odontology, Sahlgrenska Academy, University of Gothenburg, Gothenburg, Sweden; ^3^ Department of Pediatrics, Institute of Clinical Sciences, Sahlgrenska Academy, University of Gothenburg, Gothenburg, Sweden; ^4^ Department of Pediatrics, NU Hospital Group, Uddevalla, Sweden; ^5^ Department of Pediatric Rheumatology and Immunology, Queen Silvia Children’s Hospital, Gothenburg, Sweden; ^6^ Department of Pediatric Pneumology, Immunology and Critical Care Medicine, Charité Universitätsmedizin Berlin, Deutsches Rheuma-Forschungszentrum (DRFZ), Institute of the Leibniz Association, Berlin, Germany; ^7^ Division of Inflammation and Infection, Department of Biomedical and Clinical Sciences, Faculty of Medicine, Linköping University, Linköping, Sweden; ^8^ Unit of Rheumatology, Sahlgrenska University Hospital, Gothenburg, Sweden; ^9^ Division of Chemical Biology, Department of Life Sciences, Chalmers University of Technology, Gothenburg, Sweden

**Keywords:** CNO, CRMO, autoinflammation, neutrophils, TNFα, ROS, adalimumab

## Abstract

We describe a female patient suffering from severe chronic non-bacterial osteomyelitis (CNO) with systemic inflammation and advanced malnutrition and complete deficiency of myeloperoxidase (MPO). CNO is a rare autoinflammatory bone disorder associated with dysregulation of the innate immune system. MPO deficiency is a genetic disorder with partial or complete absence of the phagocyte peroxidase MPO. MPO deficiency has no established clinical phenotype but reports indicate increased susceptibility to infection and chronic inflammation. The patient’s symptoms began at 10 years of age with pain in the thighs, systemic inflammation and malnutrition. She was diagnosed with CNO at 14 years of age. Treatment with nonsteroidal anti-inflammatory drugs, corticosteroids, bisphosphonates or IL1-receptor antagonists (anakinra) did not relieve the symptoms. However, the patient responded instantly and recovered from her clinical symptoms when treated with TNFα blockade (adalimumab). Three years after treatment initiation adalimumab was withdrawn, resulting in rapid symptom recurrence. When reintroducing adalimumab, the patient promptly responded and went into remission. In addition to clinical and laboratory profiles, neutrophil functions (reactive oxygen species, ROS; neutrophil extracellular traps, NETs; degranulation; apoptosis; elastase activity) were investigated both in a highly inflammatory state (without treatment) and in remission (on treatment). At diagnosis, neither IL1β, IL6, nor TNFα was significantly elevated in serum, but since TNFα blockade terminated the inflammatory symptoms, the disease was likely TNFα-driven. All neutrophil parameters were normal both during treatment and treatment withdrawal, except for MPO-dependent intracellular ROS- and NET formation. The role of total MPO deficiency for disease etiology and severity is discussed.

## Introduction

There are several rare monogenic autoinflammatory bone disorders, e.g., deficiency of interleukin-1 receptor antagonist (DIRA, affected gene *IL1RN*), Majeed syndrome (affected gene *LPIN2*), cherubism (affected gene *SH3BP2*), as well as the recently described disease LIRSA/CRMO3 (affected gene *IL1R1)* (1–5). The pathogenesis of several of these early-onset diseases involve the IL-1 pathway and it is suggested that targeted therapies with IL-1 inhibitors should be effective in these patients (1, 2, 4). Most cases of autoinflammatory bone disorders are however polygenic, or multifactorial, with unknown pathophysiology. These are generically termed chronic non-bacterial osteomyelitis (CNO) and are characterized by dysregulation of the innate immune system, causing inflammation in sterile bone, typically affecting metaphysis of the long bones (6–8). CNO is considered a very rare disease for which the prevalence has been estimated to between 1/160 000 and 1/2 000 000 (9). Regarding nomenclature, several different terms have been proposed for this group of diseases: non-bacterial osteitis (NBO), chronic recurrent multifocal osteomyelitis (CRMO), and synovitis, acne, pustulosis, hyperostosis, and osteitis (SAPHO) (6, 8, 10–12). Recently, CNO was proposed to be the primary denomination (12) and is thus used in this report. In polygenic autoinflammatory bone disorders genetic analyses are complex and attempts to understand the pathophysiology rely primarily on cellular and cytokine analyses. It has been suggested that an imbalanced cytokine expression by monocytes such as reduced IL10 and IL19 (anti-inflammatory cytokines) and increased IL1β, TNFα, IL6 and IL20 (pro-inflammatory cytokines), drive osteoclast differentiation and bone inflammation in these disorders (13). In addition, a clinical response to blockade of pro-inflammatory cytokines (TNFα or IL1) has contributed to an increased understanding of the disease mechanism(s) in these multifaceted syndromes (13–16).

Myeloperoxidase (MPO) deficiency is a condition characterized by low (more common) or absent (much less common) levels/activity of this phagocyte peroxidase. The overall prevalence of MPO deficiency differs between reports from different geographic areas; in Great Britain it is around 1 in 1000 (17), while it is much more rare in Japan and Italy (18, 19). Partial MPO deficiency is considerably more common than total MPO deficiency which comprises around 25% of all cases (17, 19). *In vitro* studies have shown that MPO deficiency affects neutrophil function, seen as impaired killing capacity of ingested microbes (e.g., *Candida albicans*) due to lack of toxic metabolites (HOCl) that normally result from the MPO-catalyzed reaction between reactive oxygen species (ROS) and halides (20, 21), as well as a compromised ability to produce neutrophil extracellular traps (NETs) (22–24). Clinically, the common understanding has been that MPO deficiency is associated with surprisingly few pathological consequences, except if combined with diabetes mellitus which leads to increased susceptibility to fungal infections (20, 21, 25, 26). This view is however challenged by a study of 100 individuals with partial or total MPO deficiency that indicates a protective effect against cardiovascular damage, but also increased incidence of severe infections and chronic inflammatory conditions (27). The presence of an enhanced inflammatory response in MPO deficiency is supported by animal studies that demonstrate increased levels of pro-inflammatory cytokines upon phagocytic (zymosan) stimulation in MPO^-/-^ mice (28, 29). Recent data also indicate an important role for MPO in bone homeostasis (30). Hence, there is a need to further study MPO deficiency, partial as well as total, in human non-infectious (e.g., CNO) and infectious inflammatory conditions to better understand the overall role of MPO.

This report describes a patient suffering from both CNO and total MPO deficiency. Whether the MPO deficiency contributes to the CNO symptoms is elusive, but the fact that therapeutic TNFα blockade completely alleviated the clinical symptoms of this patient strongly indicates that TNFα is a component in the pathway(s) that drives the CNO pathology.

## Materials and methods

Clinical parameters presented in **Table 1**, including cytokines (TNFα, IL1β, IL6 and IL18), were measured by standard clinical laboratory techniques at the Clinical Immunology Laboratory at Sahlgrenska University Hospital, Gothenburg, Sweden, and levels were evaluated by comparison to established clinical reference values. Neutrophil functions were analyzed at the Phagocyte Research Laboratory, Department of Rheumatology and Inflammation Research, Gothenburg University, Sweden.

**Table 1:** Clinical characteristics.

	– adalimumab		+ adalimumab	
	~ 14 years^b^	~ 17 years^c^	~ 17 years^c^	~ 23 years^d^
Cell counts (normal range) x 10^9^/L				
WBC^a^ (4.5 - 13.0)	7.3	8.3	5.5	7.9
Neutrophils (1.8 - 8.0)	3.6	6.1	2.6	4.7
Lymphocytes (1.2 - 5.2)	3.6	1.6	2.4	2.4
Monocytes (0.1 - 1.0)	0.04	0.2	0.1	0.7
Eosinophils (0.04 - 0.40)	0.4	0.2	0.2	0.1
Basophils (0.0 - 0.1)	0.04	0.0	0.0	0.1
Thrombocytes (150 - 350)	732	478	378	425
Inflammatory markers (normal range)				
ESR (<20) mm/h^e^	80	63	16	49
CRP (<1) mg/L	54	6	<1	<1
SAA (<11) mg/L	>600	180	<11	20
TNFα (<20) pg/mL^f^	9.5	620	540	2600
IL1β (<5) pg/mL	5	<5	<5	<5
IL18 (140 - 500) pg/mL	NA	260	220	200
IL6 (<5) pg/mL	10	4.7	<2	<2
Other parameters (normal range)				
Hemoglobin (120 - 160) g/L	93	120	115	121
MPO (1 - 2 µg/5x10^4^ neutrophils)^g^	NA	0	0.16	0

a WBC, white blood cells; ESR, erythrocyte sedimentation rate; CRP, C-reactive protein; SAA, serum amyloid A; TNFα, tumor necrosis factor α; IL, interleukin; MPO, myeloperoxidase; NA, not available.

b Time of CNO diagnosis was ~ 5 months prior to initiation of treatment with adalimumab.

c At the age of 17 years, samples were drawn ~ 9 weeks after withdrawal (– adalimumab) and ~ 3 weeks after reintroduction of treatment (+ adalimumab).

d At age 23 years, the absolute thrombocyte count was measured 10 days before blood sampling for experimentation.

e The ESR value was measured twelve weeks (as compared to one week for all other parameters) after reintroduction of treatment, as this parameter is slow in recovery.

f TNFα was determined by an immunoassay (IMMUNLITE^®^ 1000, Siemens), that measures total TNFα, i.e., including also TNFα bound to adalimumab, at the accredited Clinical Immunology Laboratory, Sahlgrenska University Hospital, Gothenburg, Sweden.

g Normal range represents MPO-values of adult control neutrophils (n=3) run in parallel.

Neutrophils were isolated as previously described (31, 32). MPO content in neutrophil homogenates was analyzed by ELISA (Immunology Consultants Laboratory Inc., Portland, OR, USA) as described (33). Isolated neutrophils were measured for intra- and extracellular production of NADPH-oxidase derived ROS after stimulation with phorbol 12-myristate 13-acetate (PMA, 50 nM; Sigma, St Louis, MO, USA) using three different methods; luminol- or isoluminol-amplified chemiluminescence (CL), 2′,7′-dichlorofluorescin diacetate (DCFDA; Molecular Probes/Invitrogen, Grand Island, NY, USA), and p-hydroxyphenyl acetate (PHPA, Sigma), as described (34, 35). NET-formation was evaluated by staining with Sytox Green (Molecular Probes, Stockholm, Sweden) as described (22). Elastase activity of neutrophil lysates (corresponding to 3125 cells/well) was measured as described (23). Neutrophil apoptosis was evaluated by flow cytometry (Accuri C6, Becton Dickinson, San Jose, CA, USA) after incubation (37°C, 20 h) with buffer, LPS (100 ng/ml; Sigma) or FASL (anti-CD95-ab, 10 μg/ml; Nordic Biosite, Täby, Sweden) (36, 37). Neutrophils treated with or without TNFα (10 ng/mL, 37°C, 20 minutes; Sigma) were evaluated for surface expression of CD11b and CD62L by addition of specific antibodies (Becton Dickinson, San Jose, CA, USA) to blood after lysis of erythrocytes concomitant with fixation of leukocytes using FACS Lysing solution (Becton Dickinson) as described (33). Expression of cytochrome *b*_558_ was measured by a specific antibody (MBL International Corporation, Woburn, MA, USA) staining on isolated neutrophils.

The study was approved by the Regional Ethical Review Board in Gothenburg, Sweden (439–06). Written informed consent was obtained from the parents and/or patient and healthy adult controls at all blood samplings, in accordance with the declaration of Helsinki.

## Results

**Figure 1:**
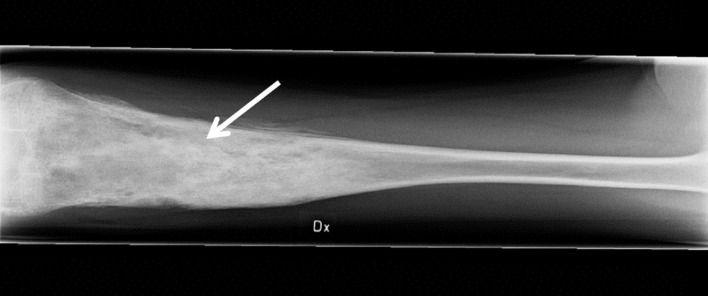
Inflammation of the femur. The anteroposterior radiograph is indicative of osteomyelitis (arrow) in the right (Dx) femur of the patient at age 14 years, prior to adalimumab treatment.

The female patient was healthy until the age of 10 years when she developed bilateral swollen and painful thighs and systemic inflammation that gradually resulted in advanced malnutrition. During a four-year period in which the family moved between countries, investigations including biopsies and magnetic resonance imaging were performed by different medical teams and the patient was subsequently diagnosed with CNO. At the time of diagnosis, the patient was 14 years old and suffered from severe malnutrition with an iso body mass index (isoBMI) of 10.5 kg/m^2^ (reference value at age 14 years; 16-26 kg/m^2^), anemia (Hb 93 g/L), and thrombocytosis (732 x 10^9^/L). Systemic inflammatory markers were elevated with an erythrocyte sedimentation rate (ESR) of 80 mm/h, C-reactive protein (CRP) of 54 mg/L, and serum amyloid A (SAA) >600 mg/L (for reference values, see **Table 1**). White blood cell count (including differential) and TNFα in serum were within normal range, whereas IL1β was minimally elevated and IL6 was slightly raised (**Table 1**). S-alkaline phosphatase was marginally increased (4.4 µkat/L; reference range <3.1 µkat/L) and antinuclear antibodies (ANAs) displayed borderline positive titers. The patient was negative for rheumatoid factor (RF), chest X-ray was normal and interferon γ-assay (QuantiFERON^®^) was negative. X-ray examination showed bilateral inflammation of the femur (**Figure 1**) and a bone biopsy indicated unspecific inflammation without granuloma. Cultures from the bone biopsy and bacterial PCR analyzed by 16S rDNA sequencing were both negative. Bone scintigraphy showed two additional loci with suspected inflammation in the left clavicle and the right acetabulum. Based on a low neutrophil signal in a Phagoburst analysis, MPO deficiency was indicated, and diagnosis was genetically confirmed, showing as a homozygous deletion of 14bp in exon 9 of the MPO-gene (c.1555-1568del14 (ATGGAACCCAACCC) p.M519PfsX21, rs536522394), a previously described mutation in individuals with total MPO deficiency (18, 24, 38–40). Accordingly, the patient neutrophils completely lacked the MPO protein (**Table 1**, and our previous reports (22, 23)). There were no autoimmune or inflammatory diseases in first-degree relatives.

Initially, the patient was treated with nonsteroidal anti-inflammatory drugs (NSAIDs) without reduction of clinical symptoms, followed by addition of corticosteroids with only a temporary effect during treatment. As a second line of therapy the patient was given NSAIDs combined with bisphosphonate infusions (pamidronate; four doses, 1 per month, first dose 0.5 mg/kg and dose two - four 1 mg/kg) which was without effect. The first biological treatment that was then attempted was IL1 receptor antagonist (anakinra, 100 mg once daily, 4 mg/kg/day, increasing to 100 mg twice daily, 8 mg/kg/day, in total 26 days) but also this treatment was without effect.

At the age of 14.5 years the patient received treatment with TNFα blockade (adalimumab, 40 mg subcutaneously biweekly). The patient responded rapidly (within two to three weeks), with normalized inflammatory markers, resolution of clinical symptoms (visual analogue scale for pain was equal to zero) and weight gain (reaching a normal isoBMI of 19 kg/m^2^ within a year). The interval between adalimumab injections was at this point prolonged to every third week.

After approximately three years of treatment, at the age of 17 years, a joint decision between the clinicians, the patient and her family were made to withdraw adalimumab. The purpose of the withdrawal was to evaluate whether the patient would remain in remission without treatment. Nine weeks after withdrawal the symptoms returned, including pain, general malaise, and increased levels of inflammatory markers (ESR, CRP and SAA). Analysis of serum cytokines showed normal levels of IL1β, IL18 and IL6 (**Table 1**). At this time adalimumab was reintroduced and the patient once again promptly went into remission upon TNF inhibition, accompanied by decrease of inflammatory markers (**Table 1**).

Based on that TNFα blockade effectively alleviated the disease symptoms suggested that this patient’s disease was TNFα-dependent and/or -driven. This is fully compatible with the patient having normal TNFα levels in serum prior to therapy initiation (**Table 1**), an observation that has been made also in other TNFα-driven inflammatory diseases such as juvenile idiopathic arthritis (JIA) and rheumatoid arthritis (RA) (41, 42). As could be expected, the measured levels of TNFα were much higher during adalimumab treatment and also some time after treatment withdrawal (9 weeks later; **Table 1**), explained by that the assay used to measure serum TNFα levels detects both free and adalimumab-bound TNFα and that adalimumab sequesters TNFα in circulation for a substantial period of time (43).

Apart from monitoring cell counts and inflammatory markers during withdrawal and reintroduction of treatment (**Table 1**), blood was analyzed for basic neutrophil functions such as degranulation and activation of the respiratory burst.

**Figure 2:**
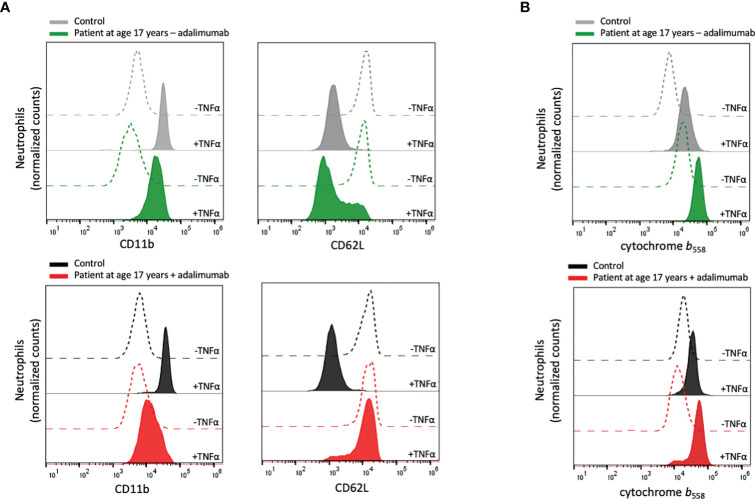
Neutrophil plasma membrane expression of CD11b, CD62L and cytochrome *b*_558_. **(A)** Peripheral blood samples from the patient and controls were incubated at room temperature without or at 37°C with TNFα (10 ng/mL) for 20 min after which the neutrophil surface expression of CD11b and CD62L was analyzed by flow cytometry. **(B)** Isolated neutrophils from the patient and controls were incubated on ice or at 37°C in the presence of TNFα (10 ng/mL) for 20 min, after which the surface expression of cytochrome *b*_558_ was evaluated by flow cytometry. Patient samples: +adalimumab (on adalimumab treatment), –adalimumab (withdrawal of adalimumab). Control samples: healthy adult donors.

Neutrophils in an inflammatory milieu undergo mobilization of intracellular granules, degranulation, which results in a more activated (primed) phenotype that can be measured as changes in cell surface markers (44, 45). To investigate whether the patient’s neutrophils displayed an activated phenotype in circulation, the expression of the surface markers CD11b and CD62L was examined in whole blood, i.e., without isolation of the neutrophils from other blood cells and plasma. The patient neutrophils displayed a resting phenotype with similar surface expression of these markers as control neutrophils, both during treatment withdrawal and after reintroduction of adalimumab (**Figure 2A**).

TNFα is a well-known priming agent for human neutrophils, and to investigate the ability of the patient’s neutrophils to respond to TNFα, recombinant TNFα was added to whole blood samples after which neutrophils were isolated and tested for stimulus-induced receptor up- and downregulation. When the analyses were performed nine weeks after adalimumab withdrawal, the patient neutrophils responded by altered surface marker exposure (**Figure 2A**), indicating that the residual level of adalimumab was not sufficient to sequester added TNFα and that the patient’s neutrophils exhibited normal regulation of TNFα-induced cell activation. In contrast, no TNFα-induced degranulation could be detected during adalimumab treatment, explained by that the levels of adalimumab in the circulation (and in the blood sample) were able to sequester the added TNFα.

Based on the above findings, we used an alternative approach to test neutrophil responsiveness to TNFα during adalimumab treatment. By isolating neutrophils from the adalimumab-containing plasma prior to TNFα treatment and then measuring cell surface levels of mobilizable markers, for this assay the cytochrome *b*_558_ was used, adalimumab-independent TNFα-induced degranulation could be investigated. The isolated neutrophils displayed increased surface expression of cytochrome *b*_558_ in response to TNFα in a similar way as the control neutrophils (**Figure 2B**), supporting that the patient neutrophils were fully capable of responding to TNFα in an *in vitro* setting.

**Figure 3:**
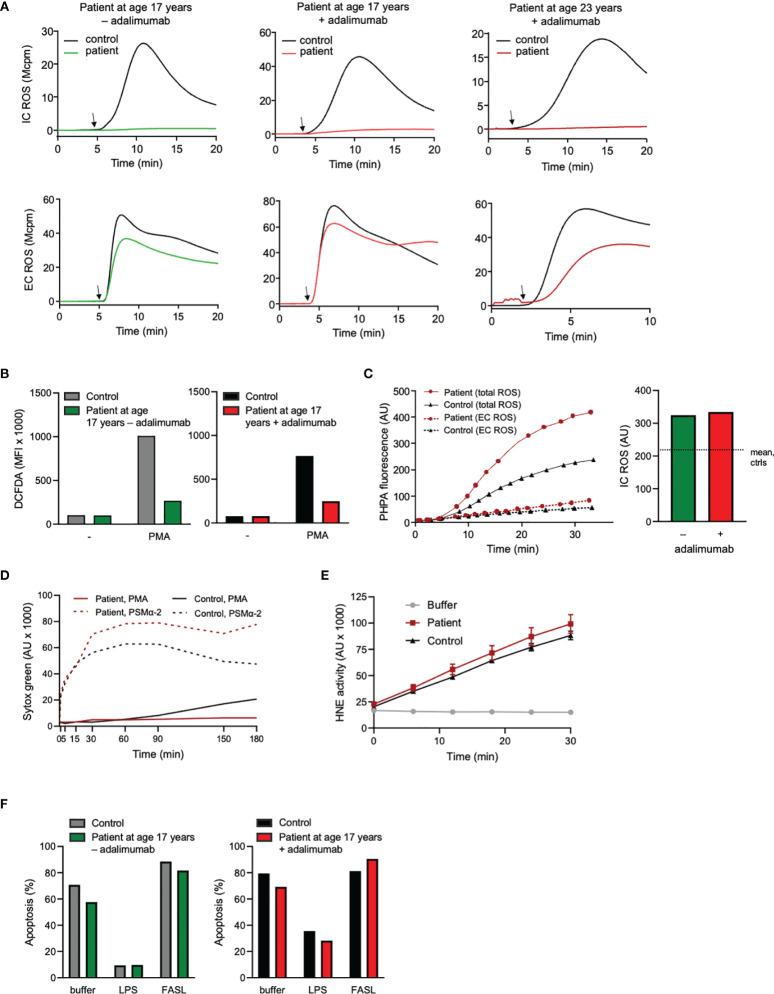
Neutrophil production and processing of ROS, NET formation, elastase activity, and viability. **(A)** Neutrophil intra (IC)- and extracellular (EC) ROS production was measured (mega counts per minute; Mcpm) by luminol- or isoluminol-enhanced chemiluminescence (CL), respectively, upon stimulation with PMA (50 nM; added at arrow after 5 min pre-incubation of cells at 37°C). **(B)** Neutrophils were stained with DCFDA for 30 min at 37°C in the absence or presence of PMA (50 nM) during the final 15 min. Thereafter ROS production was evaluated by flow cytometry. ROS production is given as geometric mean fluorescent intensity (MFI). **(A, B)** + adalimumab (on adalimumab treatment), – adalimumab (withdrawal of adalimumab). **(C)** PHPA-oxidation by hydrogen peroxide (H_2_O_2_) was used for MPO-independent evaluation of neutrophil ROS production. Neutrophils were incubated with HRP and PHPA and stimulated with PMA (50 nM) after which oxidation of PHPA was monitored by fluorometry (arbitrary units, AU) over time (min). Total H_2_O_2_ production was measured in the presence of azide (1 mM, solid lines, left graph) to abrogate H_2_O_2_ consumption by intracellular MPO and catalase, while extracellular H_2_O_2_ was measured in the absence of azide (dashed lines, left graph). The bar graph to the right shows the PHPA fluorescence representing intracellular ROS (arbitrary units, AU; calculated by subtracting the values of EC ROS from total ROS after 30 min stimulation) in the patient neutrophils (age 17 years +/- adalimumab). The horizontal dotted line shows mean IC ROS of control neutrophils after 30 min stimulation (n=2, mean 215.6 +/- SD 49.1). **(D)** To investigate the formation of NETs, neutrophils were incubated with the cell impermeable DNA stain Sytox green before stimulation with PMA (50 nM) or PSMα-2 (2.5 μM) and fluorescence (arbitrary units, AU) was monitored over time (min). **(E)** Human neutrophil elastase (HNE) was measured by incubating lysates (corresponding to 3125 neutrophils/sample) from the patient (age 23 years, + adalimumab) and one control with an elastase-specific substrate while fluorescence was monitored over time. Mean fluorescence ± SD of triplicates are shown (arbitrary units; AU). **(F)** Neutrophil apoptosis (Annexin V positive cells) was evaluated by flow cytometry after incubation of the cells with buffer, LPS (100 ng/ml) or FASL (anti-CD95 ab, 10 μg/ml) for 20 h at 37°C (number of apoptotic cells are given as fraction of total cells, %). Patient samples: +adalimumab (on adalimumab treatment), –adalimumab (withdrawal of adalimumab); Control samples: healthy adult donors.

Another neutrophil activation feature is the respiratory burst achieved by activation of the NADPH-oxidase and resulting in production of reactive oxygen species (ROS) such as superoxide anion and hydrogen peroxide. Neutrophil production of ROS was investigated using standard techniques, including isoluminol/luminol amplified chemiluminescence (CL) which can distinguish extracellularly released ROS from ROS produced at intracellular sites (34, 35), the fluorescent stain DCFDA that measures intracellular ROS-production only, and PHPA-oxidation that can measure hydrogen peroxide extra- and intracellularly. The two first techniques are dependent of MPO for the detection of intracellular ROS, and hence these assays gave an expected negative result for this parameter in the MPO-deficient patient neutrophils when stimulated with the standard agonist phorbol myristate acetate (PMA), regardless of adalimumab treatment or not (**Figures 3A, B**). The production of intracellular ROS was instead proven by PHPA-oxidation, showing normal levels in the patient cells both during withdrawal and after reintroduction of adalimumab, as compared to controls (**Figure 3C**). Extracellular release of ROS was also similar (**Figure 3A**), or even higher (**Figure 3C**), in patient cells as compared to controls, which is corroborated by other studies showing increased/prolonged production of hydrogen peroxide by MPO-deficient cells (20, 46).

MPO-processed ROS have been shown to be involved in formation of neutrophil extracellular traps, NETs, when the cells are triggered by certain stimuli, including PMA (22–24, 47). We have previously shown that prior to therapy introduction, this patient’s neutrophils were unable to form NETs in response to PMA (22) while NET formation in response to PSMα-2 peptides (ROS independent NET-inducers (23)) was extensive. We here confirmed those previously published data with novel data showing that also when symptoms were alleviated by adalimumab therapy, NET formation in response to PMA (but not to PSMα-2) was defective, in line with the MPO deficiency of the patient (**Figure 3D**). Apart from MPO-processed ROS, neutrophil elastase activity is critical for PMA-induced NET formation (48, 49) but patient neutrophil lysates obtained when the patient was on adalimumab therapy (at age 23 years) displayed normal elastase activity (**Figure 3E**).

Both inflammation *per se* and ROS in particular have been associated with changes in neutrophil viability (33, 36, 50). Yet, for this patient with CNO, neutrophil apoptosis was normal, including responses to pro- and anti-apoptotic stimulation, both during the inflammatory flare caused by treatment withdrawal and after reintroduction of treatment (**Figure 3F**), suggesting that regulation of apoptosis by ROS *per se* was unaltered in the patient leukocytes.

Taking together the data on neutrophil ROS-production, processing of ROS by MPO and induction of associated cellular functions such as NET formation and apoptosis, we conclude that NADPH-oxidase activity is overall intact in the patient neutrophils while mechanisms involving the processing of the resulting ROS, such as the formation of NETs, are impaired due to total lack of MPO. Whether the altered processing of ROS could be of importance for the pathophysiology of the patient’s disease is discussed below.

Hypothetically, a link between the total MPO deficiency and the TNFα-driven pathology could be that TNFα production is, under healthy conditions, limited by MPO-processed ROS. To attempt to test a possible dependency between MPO and TNFα we assessed the production of pro-inflammatory cytokines from overnight-incubated total isolated leukocytes (devoid of red blood cells and plasma containing adalimumab) obtained from the patient at the last sampling (age 23 years, with adalimumab treatment). However, we could not detect any altered production of TNFα, IL1β or IL8 as compared to control cells (**Supplementary Figure 1**), indicating that there is no direct link between MPO deficiency in phagocytic cells and their capacity to produce TNFα that could be of potential importance for regulating the CNO-associated systemic inflammation.

## Discussion

The patient presented in this study has a complex clinical presentation, diagnosed with CNO and total MPO deficiency together with severe systemic inflammation and progressive malnutrition. The combination of CNO and MPO deficiency has to our knowledge not been described before, and if it is a coincidence in this patient or occurs also in other cases remains to be investigated. Regardless, a question that must be posed based on this finding is whether total MPO deficiency could contribute to the etiology or severity of CNO.

At diagnosis, analysis of cytokines gave few clues as to what disease mechanisms could be responsible for the severe symptoms of the patient, showing minimally elevated serum levels of IL1β, slightly increased levels of IL6 and no increase in TNFα (**Table 1**). The patient was treated according to international recommendations, initially with NSAIDs, corticosteroids and bisphosphonate infusions (13, 14, 16). As this treatment failed, biological therapy was introduced, starting with IL1 receptor antagonist (anakinra) as this drug has a short half-life in circulation (4-6 hours) making it possible to switch to TNFα blockade without delay upon treatment failure. IL1 blockade has been shown successful in several monogenic autoinflammatory bone disorders such as DIRA, Majeed syndrome and LIRSA/CRMO3 (1, 2, 4) even in the absence of detection of IL1 in serum, however, in this patient anakinra was unsuccessful. When TNFα blockade (adalimumab) was initiated, the patient responded instantly and recovered promptly from clinical symptoms. The successful treatment with adalimumab strongly implies that TNFα drives inflammation in this patient. Previous data on serum TNFα levels in patients with CNO have been conflicting (51, 52); the patient described here showed no increased levels of TNFα in circulation at diagnosis, suggesting that the TNFα-driven pathogenesis was of local origin but causing systemic inflammation (see below).

The described patient displayed a total lack of neutrophil MPO. Since CNO is rarely associated with such severe systemic inflammation and advanced malnutrition as seen in this patient, we were prompted to ask if and how the MPO deficiency may contribute to the disease mechanism of this patient. Such an hypothesis gains support from published data; cells from mice deficient in MPO produce increased levels of cytokines including TNFα (28, 37), as do cells that cannot produce NADPH-oxidase-derived ROS, isolated from patients suffering from chronic granulomatous diseases (CGD) (53). In fact, patients with MPO deficiency or CGD both endure chronic inflammatory conditions (27, 54), supporting a possible role for MPO in aggressive CNO. The phenotype presented by the patient could be influenced by other inflammation-driving cell types such as monocytes. Since total MPO deficiency is characterized also by lack of MPO in monocytes, an alteration of monocyte function being part of the pathophysiology is possible and merits consideration.

In neutrophils, which normally contain high levels of the enzyme, MPO is responsible for processing NADPH-oxidase-derived ROS into highly microbicidal metabolites such as hypochlorous acid (55). Lack of MPO would thus suggest an impaired microbial killing, in line with that MPO deficiency was originally associated with *Candida* infection in diabetic patients (21, 56), even though the majority of affected individuals were clinically healthy (20, 21, 57). However, in a study of 100 individuals with subtotal or total MPO deficiency, Kutter et al. in fact demonstrated an increased incidence of severe infections as well as chronic inflammatory conditions (27). In the patient described here, who displayed total MPO deficiency, there were no indications of increased frequency of infection, suggesting compensatory mechanisms to the specific MPO function that provides retained antimicrobial activity in the phagolysosome when MPO is absent (20, 51).

The central role of TNFα in driving the severe systemic inflammation in the patient presented here was proven by the responsiveness to anti-TNFα therapy. The disease was however not associated with increased levels of TNFα in circulation at presentation, yet with a substantial local inflammation in the bone, evident by the x-ray, bone biopsy and high acute phase reactants in serum, which signals that a full inflammatory cascade reaction is taking place. This suggests that local TNFα production might drive the systemic inflammation. One possible explanation could be that the MPO deficiency confers a loss of the protective role that MPO normally has on bone turnover through controlling osteoclastogenesis and bone resorption (30). In fact, MPO deficiency in mice potentiated the RANKL‐initiated signal transduction activation including NF‐κB among other transcription factors which led to production of pro-inflammatory cytokines, possibly enhancing the inflammatory cascade locally and systemically. Interestingly, a recent publication shows that MPO deficiency can also be associated with severe forms of neutrophil dermatosis, i.e., generalized pustular psoriasis and palmoplantar psoriasis (58).

Another contributing mechanism could be the MPO-dependent formation of NETs. Aggregated NETs promote the resolution of neutrophilic inflammation by proteolytic degradation of cytokines and chemokines via serine proteases such as elastase, and thereby disrupt an ongoing inflammatory recruitment and activation of leukocytes (59). In a situation where a local inflammation is not limited by clearance of cytokines through formation of NETs (e.g., due to MPO deficiency) but instead end up in a pro-inflammatory self-feeding loop, as indicated in the bone of the presented patient, it is possible that a normally more contained inflammatory disease such as that seen in CNO may erupt into a severe inflammatory condition as that described here.

The wide variety in clinical consequences associated with MPO deficiency, from none to quite severe, demands for further epidemiological studies to be undertaken. Further, it is important to keep in mind that the generalizations on clinical consequences of MPO deficiency are most probably made based on the larger population of patients with *partial* MPO deficiency. Pathologies associated with the rarer condition of total MPO deficiency may be much less apparent in a cohort material. Such pathologies might include autoinflammatory syndromes such as CNO, where a co-occurrence of total MPO deficiency possibly could lead to triggering CNO disease or perhaps increase the severity of “regular CNO”, as in the patient described here. Regardless of the disease mechanism and the role for total MPO deficiency, it may be of great value to the clinician that encounters severe CNO or other inflammatory conditions with an unexpected severe phenotype to test for total MPO deficiency and to explore the clinical response to different cytokine blockers in a structured manner, especially TNFα blockers that was of such importance for this patient.

## Concluding remarks

In this study we describe a patient with severe CNO, malnutrition, systemic inflammation and total MPO deficiency. The patient was successfully treated with TNFα blockade resulting in instant resolution of the inflammatory symptoms disclosing that the disease was TNFα-driven. However, the treatment did not permanently abolish the underlying inflammatory condition, as the symptoms returned upon treatment withdrawal. After reintroduction of treatment the patient returned into remission.

Neutrophils from the patient during withdrawal and after reintroduction of treatment did not differ substantially with regards to NADPH-oxidase activation, viability or surface marker exposure as compared to cells collected during treatment. However, the absence of MPO-dependent NET-formation was evident at all times.

We speculate that the total MPO deficiency aggravates the TNFα-driven inflammation in this case of CNO, both by causing a loss of the MPO-mediated protective effect on bone turnover and by our shown deficiency in NET-formation that could lead to chronic local neutrophilic inflammation with resulting systemic manifestations. The molecular and clinical consequences of total MPO deficiency in an autoinflammatory setting need to be more thoroughly investigated. Also, to what extent a decreased capacity to form NETs could explain the increased proportion of inflammatory conditions in patients with MPO deficiency needs to be explored.

## Data availability statement

The original contributions presented in the study are included in the article/**Supplementary Material**. Further inquiries can be directed to the corresponding author.

## Ethics statement

The studies involving human participants were reviewed and approved by The Regional Ethical Review Board in Gothenburg, Sweden. Written informed consent to participate in this study was provided by the participant's legal guardian/next of kin and the participant.

## Author contributions

The patient was under the care of TK, SB and PW. The experiments and analyses were performed by MS, KC, HB, FS, AD and AW with input from JB, LB and AK-B. MS and KC drafted the manuscript which was then critically revised by all authors before approval of the final version to be published. All authors contributed to the article and approved the submitted version.
