# Supplementary material for: Severe chronic non-bacterial osteomyelitis in combination with total MPO deficiency and responsiveness to TNFα inhibition
Source: Front Immunol. 2023 Oct 26;14:1233101. doi: 10.3389/fimmu.2023.1233101 (PMC10637399; doi:10.3389/fimmu.2023.1233101)
Supplement: Supplementary file 1 [file Image_1.pdf]

Supplementary Figure 1, Sundqvist, Christenson *et al*

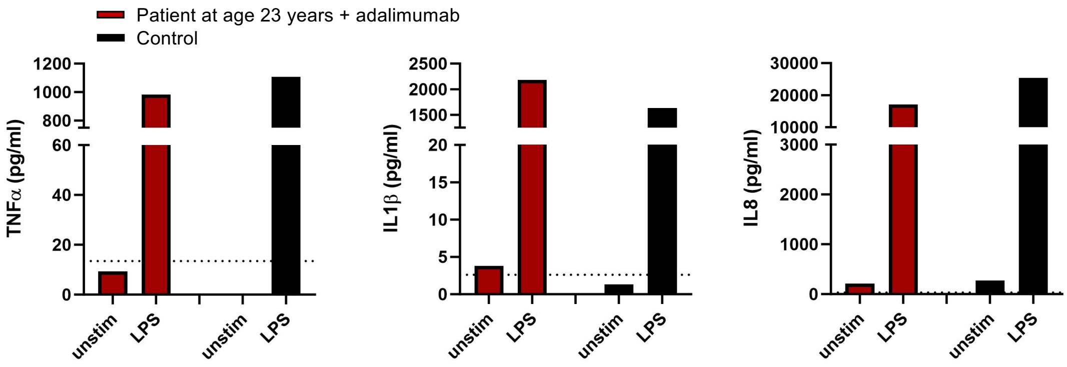

Supplementary Figure 1.

Total leukocytes isolated from the patient at age 23 years (when under treatment with adalimumab) and from a healthy control were incubated for 20 h with or without LPS (100 ng/mL). The production/release of TNFα, IL1β and IL8 were measured in the supernatants with ELISAs. The TNFα (#DY210) and IL8 (#DY208) ELISAs were from R&D systems and the IL1β ELISA (#437004) was from Biolegend.
